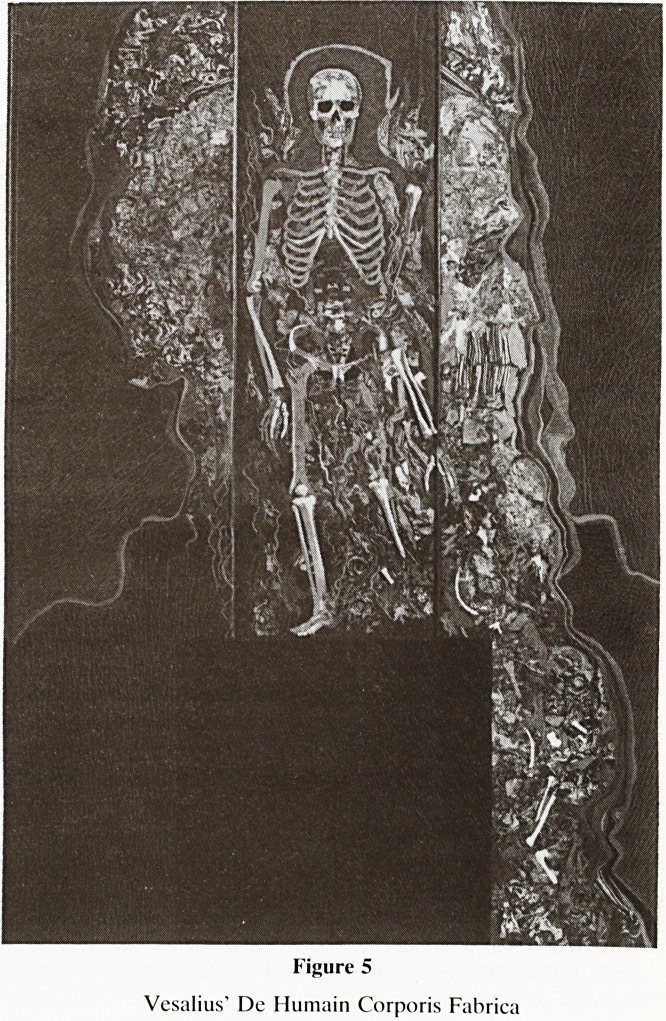# Leather

**Published:** 1989-11

**Authors:** J. M. A. Smith

**Affiliations:** Medical Student, Bristol University


### Leather

**J. M. A. Smith**
Medical Student, Bristol University

Leather is derived from the hides of skins of animals. It is a remarkably durable, strong and versatile material, which in its various forms has been made into objects ranging from African shields and drums to European saddles and kinky underwear. From as early as 2700 BC until fairly recently leather was also in the forefront of information technology with its use (as skins, vellum and parchment), for writing on, and its important role in bookbinding. (Uses of leather; Figure 1).

The skins usually used are derived from cattle, goats, pigs and sheep as byproducts of the meat industry, though theoretically any skin can be used from ostrich to sharkskin or snakeskin. Closer to home, in the medical school pathology museum at Bristol University stands the skeleton of a condemned criminal, whose skin was apparantly used to bind his legal case notes.

Essentially leather consists of the modified dermis of the skin (Figure 2). The dermis, or corium in leathermaking terms, consists of the grain layer (papillary dermis) and the fibre network layer. It consits of four major components: collagen, elastin, ground substance, and the cellular components.

It is the first of these, collagen, which composes 80–85% of the dermis by dry weight, which forms the reticular network or weave of fibres, imparts much of its bulk and strength to the skin, and gives leather a unique character which to date has been impossible to reproduce artificially. The complex and random manner in which the fibres ramify is interrupted in the grain layer by the hair follicles, giving rise to the characteristic pattern or grain unique to each animal and so prized by leatherworkers. So deeply engrained (!) in human nature is the attraction afforded by the textured surface, that artificial grains may be embossed upon plain surfaces of split skins, on defective hides to give an impression of better quality, and even on artificial materials such as plastics.

**Figure 1**

Uses of leather.

**Figure 2**

Structure of skin and leather

**Figure 3**

Factors influencing properties of animal skins and leathers

During life, skin is a dynamic structure. There is of course constant turnover of the epidermis, with an epidermal transit time of 28 days, but there is also continuous recycling and renewal of the collagen network, as well as of elastin and the stromal ground substance, by the cellular components of the skin.

With increasing age the skin of an animal increases in thickness, but the ratio between the grain layer and the fibre network layer of the corium remains constant. Since the collagen network in the grain layer is interrupted by the hair follicles it follows that the grain layer is weaker than the rest of the corium. Coupled with the fact that there is a net decrease in the amount of collagen (as well as elastin) with age, it may be seen that the skin of a young animal makes a finer, stronger leather than that of an adult which has been pared down to make a leather of equivalent thickness (see figure 3).

Other factors influencing the quality of the products that can be made from leather include the species, sex, health and diet of the animal, and the site from which the leather was taken (Figure 3).

In essence, the manufacturing process can be divided into three stages: pretanning, tanning and finishing (post tanning). However each skin may have to undergo a plethora of steps (Table 1), the exact nature and order of which will depend upon the type of skin, and the intended use of the finished product.

Pretanning involves the removal of the skin or hide from the animal, preserving, softening, and preparation for the subsequent tanning. The unwanted subcutaneous fat, and the hair and epidermis must be removed by a mixture of mechanical and chemical methods, and the fibre network of the corium must be loosened or otherwise exposed to the action of tans, by the removal of varying fractions of the ground substance.

Once upon a time, these preliminary steps required the use of such antisocial raw materials as dog or chicken dung (which supplies the enzyme trysin and aids softening of the hide and loosening of the epidermis and hair), and the beating in of oils to facilitate the tanning. The process has been developed in modern times to be rather less prolonged and a great deal less unpleasant by the use of powdered enzyme mixtures, chemicals and of course mechanization.

The tanning that follows is no less complicated. There are many different types of tan (Table 1), each imparting slightly different properties to the leather, though most have actions in each of the following spheres:

1. Increasing the number of stable bonds between collagen molecules, thus strengthening it and resisting bacterial breakdown, and oxidation.
2. Increasing the number of crosslinks between natural and added oils again increasing durability of the skin while maintaining the suppleness.
3. Filling the spaces between the collagen weave, reducing elastic recoil and enabling maintenance of the distortion caused by tooling and moulding.
4. Action of ingredients in the tanning mixture as buffers reducing damage due to atmospheric pollutants such as sulphurdioxide and exposure to human sweat etc. Other agents may directly inhibit bacterial growth.

Once tanning is completed the leather undergoes a variety of further processes (Table 1), before it is ready for its intended use.

The prayer book (Figure 4) shows the interesting use of leather to recreate the texture of skin on the hands (balsa wood and epoxy putty sculptured bookboards), and, the illustration of the binding of Vesalius' De Humani Corporis Fabrica, a magnificent collection of early, but highly accurate and ahead of their time, anatomical studies (Figure 5) shows the comparative anatomy (grains) of the skins of different

**Table 1**
**Processes in Leather Manufacture**

| *Process* | *Function* |
|---|---|
| *Pre-tanning* | |
| Flaying | produces raw hide or skin from carcass |
| Curing | prevents decay |
| Soaking | cleans and softens |
| Liming | loosens hair and epidermis |
| Unhairing and Scudding | removes hair and epidermis |
| Fleshing | removes subcutaneous tissues |
| Splitting | produces a greater surface area and produces leathers for different uses |
| Deliming | neutralizes alkalinity |
| Bating | enzymatic softening and loosening of fibre network weave |
| Pickling | preserves, de-greases and corrects pH |
| *Tanning* | |
| Vegetable tans | tannins produce firm, mouldable leather good for bookbinding |
| Chrome tans | gives a soft, but less flexible leather which takes dyes well |
| Zirconium tans | produces white washable leather |
| Alum tawing | soft white leather, unstable to washing and resilient to tooling |
| Oil tanning | soft, stretchy leather often used for chamois leathers |
| Aldehyde tanning | produces a washable leather with similar properties as vegetable tans |
| Smoke tanning | forms aldehyde crosslinks to make durable skins used to improve the penetration of vegetable tans |
| *Post-tanning* | |
| Drying/Draining/Washing/Neutralizing | |
| Shaving | renders the thickness uniform |
| Dyeing | surface dyes/colouration or immersion dyes |
| Fatliquoring | emulsified oils give greater flexibility and softness |
| Setting out | removes creases which may dry in |
| Boarding | brings out natural grain qualities |
| Plating/Embossing | imparts many kinds of 'grain' patterns |
| Brushing | polishes the grain |

**Figure 4**

'Philip Smith Hands' New Testament and Psalms

**Figure 5**

Vesalius' De Humain Corporis Fabrica continued on p 114

LEATHER
continued from p 111

animals and different sites of origin for the leather, as well as the integration of ancient (leather) and modern (plastic Airfix model skeleton) materials.

The advent of modern methods and synthetic materials has, as in most fields including medicine, greatly affected utilizaton of older methods and materials. But, while the utilization of artificial polymer based materials has intruded substantially into the realm of footwear and the widespread adoption of the paperback book has caused the decline of leather in another very different field, the occurrence of unforeseen complications such as Juvenile Plantar Dermatosis due to the former, and the decline of traditional bookbinding with the new trend towards the artist craftsman, makes such leather products all the more desirable, and it seems unlikely that this very complex and useful substance will ever by entirely replaced.

**ACKNOWLEDGEMENTS**
I would like to thank Dr. J. R. Burton for his encuragement and Mr. C. P. Smith for the kind loan of photographs of his bookbindings.

**BIBLIOGRAPHY**
REED, R. 1972. Ancient Skins, Parchments and Leathers. Seminar Press. London and New York.
HAINES, BETTY M. 1987. Book Binding Leather. The New Bookbinder. Vol. 17, pp 63–82.
Progress in Leather Science 1920–1945. pp 426–451. BLMRA: London 1948.
HOOVER, E. M. Jr. 1937. Location Therapy and the Shoe and Leather Industries. Harvard University Press.

## Figures and Tables

**Figure 1 f1:**
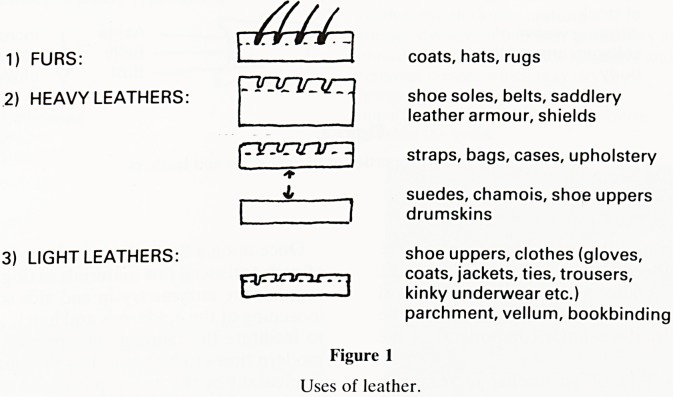


**Figure 2 f2:**
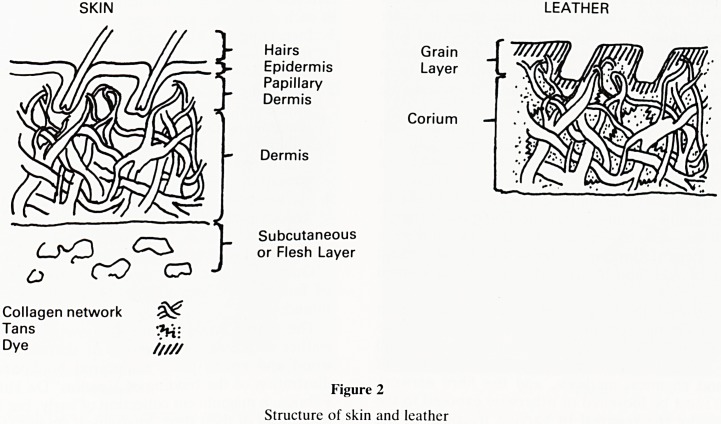


**Figure 3 f3:**
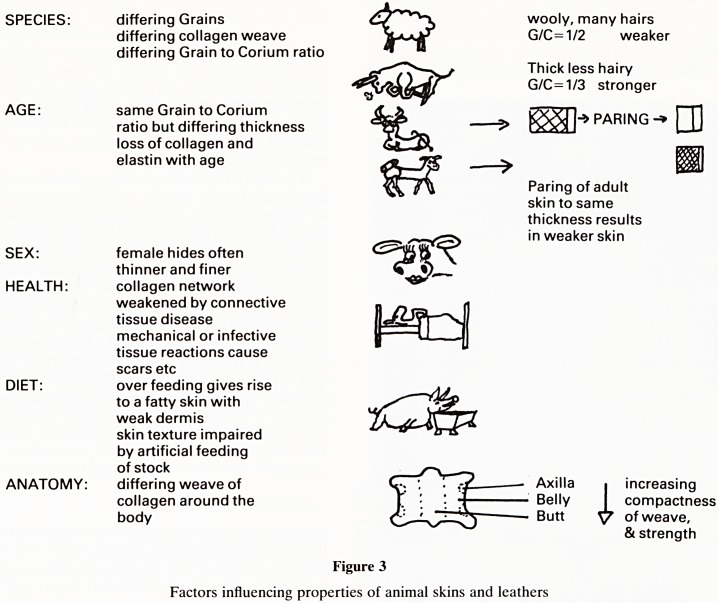


**Figure 4 f4:**
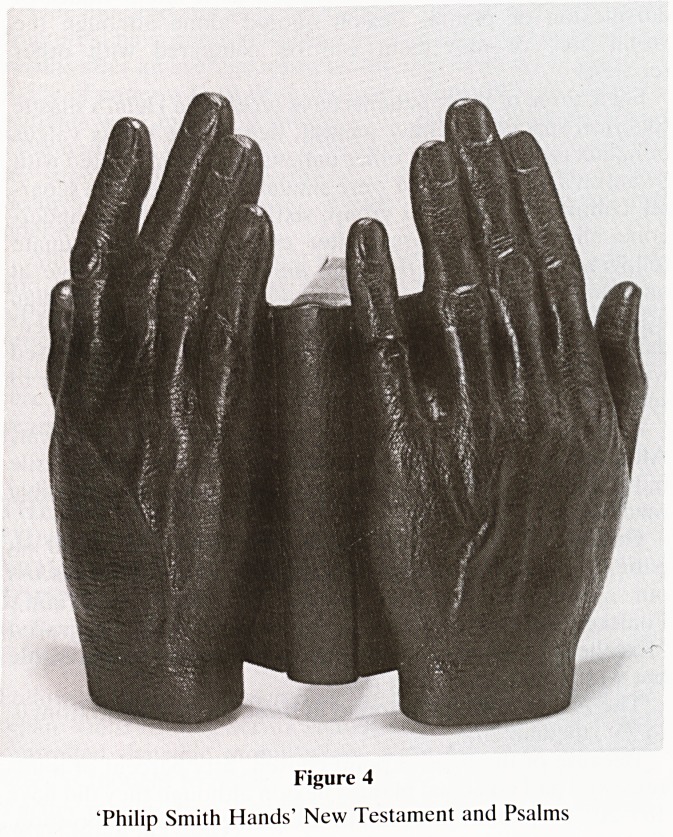


**Figure 5 f5:**